# Leveraging User-Centered Design and Usability and Skills Testing for a Novel Diabetes Survival Skills Toolkit

**DOI:** 10.1210/jendso/bvaf111

**Published:** 2025-06-24

**Authors:** Stephen Freeman, Liam Lecka, Ka’Derricka Davis, Grace Prince, Kevin Carthy, Jane Jeffrie Seley, Jing Song, Jungwha Lee, Stacy Cooper Bailey, Rebeca Khorzad, David Gatchell, Bruce Ankenman, Daniel Rees Lewis, Jane L Holl, Amisha Wallia

**Affiliations:** Division of Endocrinology, Metabolism and Molecular Medicine, Northwestern University Feinberg School of Medicine, Chicago, IL 60611, USA; Center for Health Services & Outcomes Research, Northwestern University Feinberg School of Medicine Institute for Public Health and Medicine, Chicago, IL 60611, USA; Center for Health Services & Outcomes Research, Northwestern University Feinberg School of Medicine Institute for Public Health and Medicine, Chicago, IL 60611, USA; Division of Endocrinology, Diabetes and Metabolism, Virginia Commonwealth University, Richmond, VA 23298, USA; Division of Endocrinology, Metabolism and Molecular Medicine, Northwestern University Feinberg School of Medicine, Chicago, IL 60611, USA; Division of Endocrinology, Diabetes and Metabolism, Weill Cornell Medicine, New York, NY 10021, USA; Department of Preventative Medicine, Division of Biostatistics and Informatics, Northwestern University Feinberg School of Medicine, Chicago, IL 60611, USA; Department of Preventative Medicine, Division of Biostatistics and Informatics, Northwestern University Feinberg School of Medicine, Chicago, IL 60611, USA; Division of General Internal Medicine, Northwestern University Feinberg School of Medicine, Chicago, IL 60611, USA; Department of Industrial Engineering and Management Sciences, Northwestern University McCormick School of Engineering, Evanston, IL 60208, USA; Department of Biomedical Engineering, Northwestern University McCormick School of Engineering, Evanston, IL 60208, USA; Department of Industrial Engineering and Management Sciences, Northwestern University McCormick School of Engineering, Evanston, IL 60208, USA; Department of Learning Sciences, Northwestern University School of Education and Social Policy, Evanston, IL 60208, USA; Center for Healthcare Delivery Science and Innovation, University of Chicago, Chicago, IL 60637, USA; Division of Endocrinology, Metabolism and Molecular Medicine, Northwestern University Feinberg School of Medicine, Chicago, IL 60611, USA; Center for Health Services & Outcomes Research, Northwestern University Feinberg School of Medicine Institute for Public Health and Medicine, Chicago, IL 60611, USA; Department of Preventative Medicine, Division of Biostatistics and Informatics, Northwestern University Feinberg School of Medicine, Chicago, IL 60611, USA

**Keywords:** diabetes mellitus, diabetes self-care education, remote education delivery, systems usability scale, user-centered design, user testing

## Abstract

**Purpose:**

With the expansion of telemedicine, patient-centered approaches for delivering diabetes mellitus (DM) self-care education in both in-person and remote settings are needed. A novel Diabetes Survival Skills Toolkit (Kit) (physical toolkit, website, paper guide) was developed, using a user-centered design approach. The aim of this study was to develop a hybrid protocol to assess the perceived usability of the Kit and the skills attainment of its users.

**Methods:**

Adults without prior exposure to DM self-care were recruited. User tests were conducted between January 2021 and July 2022. Initially, the usability of the website alone was tested. Then, usability and skills attainment tests were conducted with all 3 components delivered together. Usability was measured by the System Usability Scale (SUS) and skills attainment was measured thorough simulated insulin injection and lancing device use.

**Results:**

User tests (N = 43) were conducted remotely (27/43; 63%) and in-person (16/43; 37%). SUS scores were largely excellent (35%) or acceptable (47%). Users who completed skills attainment testing (N = 32) all successfully injected insulin with simulation supplies. However, SUS scores and skills attainment were poorly correlated: users with unacceptable SUS scores (4/32, 13%) successfully attained the tested skills, while 2 of the 3 users who did not demonstrate successful lancing device use had excellent SUS scores.

**Conclusion:**

Hybrid user testing of a multi-component Kit to teach DM survival skills showed high skills attainment among adult users new to DM self-care. Pairing usability and skills attainment testing can help optimize the design of DM education interventions.

Diabetes mellitus (DM) survival skills refer to the key aspects of DM self-care education required to prevent, identify, and act on glycemic emergencies. Learning DM survival skills can be difficult, especially when it requires self-injecting insulin, a high-risk medication [1]. However, time and capacity to deliver DM self-care education are considerably limited in the peri- and post-pandemic era [2, 3]. Previous studies have reported the need for multi-modal DM self-care education options, including both in-person and remote delivery options [2, 4-6]. However, remote delivery of DM self-care education has been particularly challenging. Studies have shown that older adults require significant support to overcome technology barriers to remote DM self-care education delivery [7]. Several applications and websites for remote DM self-care education have been developed, but it is unclear if using these interventions truly imparts DM skills attainment. There is also a need for DM self-care education options that are patient-centered and user-friendly [8]. Previous studies have demonstrated that (i) patients are critically lacking opportunities for self-paced, simulated, DM survival skill practice [8]; and (ii) there is a need to assess and ensure DM skills attainment following delivery of the education [9]. In light of these findings and the increasing use of telemedicine, there is a substantial need for new methods for testing both usability and skills attainment among users of DM self-care education modalities in hybrid environments.

A Diabetes Survival Skills Toolkit (Kit) was created using a user-centered design (UCD) approach to meet the above needs [10, 11]. UCD is a framework that centers engaging end-users (eg, patients, clinicians) throughout the design process to optimize adoption and engagement [10, 12]. UCD has been increasingly adopted and recommended as a best practice for the design of healthcare products and services [12]. The Kit consists of (i) a physical toolkit to organize and store DM supplies needed to perform DM survival skills, including an artificial skin to practice skills on; ii) a website that provides step-by-step instructions/videos/graphics to perform DM survival skills; and (iii) a paper guide with the same instructions/graphics as the website, but in a printed format. An in-depth UCD process, as described by Prince et al [13] and Hakimian et al [9], was used to design the Kit, which included >50 interviews with patients, clinicians, administrators, and laypersons to understand critical needs in DM education for patients newly prescribed insulin from multiple stakeholder perspectives. The present study reports the development of a hybrid protocol to test the usability and DM survival skills attainment among users of the Kit.

## Methods

There were 2 sequential testing phases. Phase 1 tested the usability of the website only using the System Usability Scale (SUS). Phase 2 tested both usability (SUS) and skills attainment using all components of the Kit. Between Phase 1 and Phase 2, modifications to the website design (ie, streamlining page navigation, clarifying the site's integration with the toolkit) were made based on Phase 1 results.

### User Recruitment

Users were recruited between January 2021 and July 2022. Users from community settings were identified through the research team's local networks and subsequent snowball sampling. Written consent was obtained from all users, and the study was approved by the Northwestern Institutional Review Board. Inclusion criteria included (i) no personal history of DM; (ii) no history of exposure to DM self-care (eg, no self-reported familiarity with the DM self-care); (iii) no prior medical training; (iv) being ≥18 years of age. Users were purposively sampled by age group (<45 years, 45-65 years, >65 years) and education level (< or ≥4-year degree). In Phase 1, younger users (age ≤65 years) and those with higher educational attainment (≥4-year degree) were recruited to assess the usability of the website because they were likely to have fewer technological barriers to use. In Phase 2, older users (>65 years) and those with lower educational attainment (<4-year degree) were purposively recruited.

### User Testing

**Figure 1.**
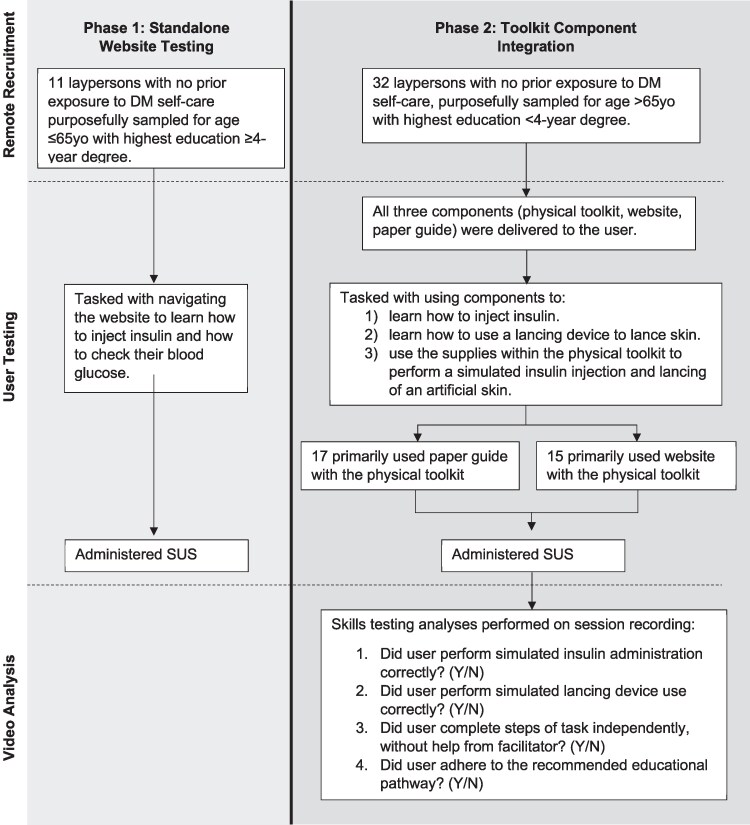
Methods graphical summary. Abbreviations: DM, diabetes mellitus; SUS, System Usability Scale.

User testing sessions were conducted by 1 of 4 trained facilitators, experienced in user-experience design. In Phase 1, usability testing of the website was conducted by video conference (Zoom). In Phase 2, users received the physical toolkit, website, and paper guide, and testing was completed either by video conference (Zoom) or in-person. User component selection was determined based on which component (website or paper guide) the user utilized to complete the majority of the steps in the test. The user testing methods used in each phase are summarized in Fig. 1.

All user tests were video recorded and followed a standard protocol. Users were provided with a clinical scenario that required them to learn to inject insulin and to use a lancing device. Each user then performed a cognitive walkthrough. This involved users describing their thought process out loud while they used the components of the Kit available to them to complete the above task [14, 15]. The trained facilitator did not provide feedback or additional instruction to the user during testing. If a user had difficulty or requested help, the facilitator waited ∼1 minute before providing minimal guidance. After completing the tasks, the SUS survey was administered (see “Usability Testing” below). Socio-demographic information, including age, highest level of education, race, and gender, were collected through a brief survey.

### Usability Testing

Perceived usability was measured by the System Usability Scale (SUS) (Appendix A) [16 ]. SUS is a validated, easily administered, and widely used 10-question survey [17-19]. User's responses were converted into a total score (maximum = 100 points), according to the methods originally described by Brooke [17]. A categorical approach was used to analyze and compare user SUS scores [20-22 ]. SUS scores were then classified as unacceptable (<65), acceptable (65 ≤ score < 85), or excellent (≥85) [20]. To identify more specific targets for improvement, users' responses to the individual SUS survey items were also analyzed [23]. Users' Likert scale responses for each SUS item were reclassified on a scale of “strongly positive” to “strongly negative” to account for the survey's alternating positively and negatively worded statements and facilitate easier comparison of mean responses between survey items.

### Skills Testing and Video Analysis

Users in Phase 2 completed skills testing in addition to usability testing. Skills testing involved users attempting to perform an insulin injection into an artificial skin and using a lancing device to lance an artificial skin to simulate obtaining a blood sample for glucose measurement. All supplies needed to inject insulin (alcohol wipes, insulin pen or insulin syringe and vial, pen or syringe needles, sharps disposal) and to use the lancing device (alcohol wipes, lancing device, lancets, sharps disposal) were provided in the physical toolkit.

Video recording and analysis methods were informed by the recommendations by Derry et al [24 ]. The full recording of each user testing session was analyzed by a facilitator who did not directly facilitate the testing session. The facilitators were trained on successful execution of the skills by a clinical expert. Four aspects of skills attainment were assessed: (i) successful simulation of insulin injection; (ii) successful simulation of using the lancet; (iii) independent execution of the skills (eg, no requirement of facilitator assistance); and (iv) adherence to the recommended educational pathway (eg, executed the steps needed to perform the skill in the order shown on the website/guide book). The clinical expert made final determinations in any cases where assessors were not certain. If a user failed to perform a simulated task, required facilitator assistance, or deviated from the educational pathway, facilitators also noted at which step of the task this occurred.

### Statistical Analysis

Statistical analysis of user data was performed using RStudio. To investigate the association between SUS-category and age-category (<45, 45-65, >65), highest education level (< or ≥ 4-year degree), and component selection (physical kit + paper guide, physical kit + website), 2-tailed Fisher's exact tests were performed.

## Results

### Sample Characteristics

A total of 43 users were recruited, of whom 27 (63%) were tested remotely (Zoom) and 16 (47%) were tested in-person (Table 1). Demographics of users are shown in Table 1, with 47% female and 77% non-Hispanic White, 14% Asian, 2% Black or African American, and 7% Hispanic. Additionally, 51% of users were <45 years old, 16% 45 to 65 years old, and 33% > 65 years old. Thirty-five percent of users had less than a 4-year degree.

**Table 1.** User socio-demographics and testing setting by component tested

	Overall (% column)	Phase 1	Phase 2	
		Website only (% column)	Tested physical kit + paper guide (% column)	Tested physical kit + website (% column)
**Number of users**	43	11	17	15
**Age, years**				
Mean (std)	46 (21.6)	34.1 (16.4)	53.9 (23.4)	45.9 (19.9)
Median	40	29	66	50
<45 years	22 (51%)	9 (82%)	6 (35%)	7 (47%)
45-65 years	7 (16%)	1 (9%)	2 (12%)	4 (27%)
>65 years	14 (33%)	1 (9%)	9 (53%)	4 (27%)
**Highest** **education level**				
<4-year degree	15 (35%)	0 (0%)	7 (41%)	8 (53%)
**≥**4-year degree	28 (65%)	11 (100%)	10 (59%)	7 (47%)
**Gender**				
Male	23 (54%)	6 (55%)	10 (59%)	7 (47%)
Female	20 (47%)	5 (45%)	7 (41%)	8 (53%)
**Ethnicity and race**				
Non-Hispanic White	33 (77%)	8 (73%)	14 (82%)	11 (73%)
Non-Hispanic Asian	6 (14%)	2 (18%)	2 (12%)	2 (13%)
Non-Hispanic Black or African American	1 (2%)	0 (0%)	0 (0%)	1 (7%)
Hispanic	3 (7%)	1 (9%)	1 (6%)	1 (7%)
**Setting**				
Virtual (Zoom)	27 (63%)	11 (100%)	7 (41%)	9 (60%)
In-person	16 (37%)	0 (0%)	10 (59%)	6 (40%)

Abbreviation: SUS: System Usability Scale.

Purposeful sampling resulted in distinct samples with younger (82% < 45 years), more educated (100% ≥ 4-year degree) users tested in Phase 1 and older (41% > 65 years), less educated (47% with <4-year degree) users tested in Phase 2.

### System Usability Scale Surveys

**Figure 2.**
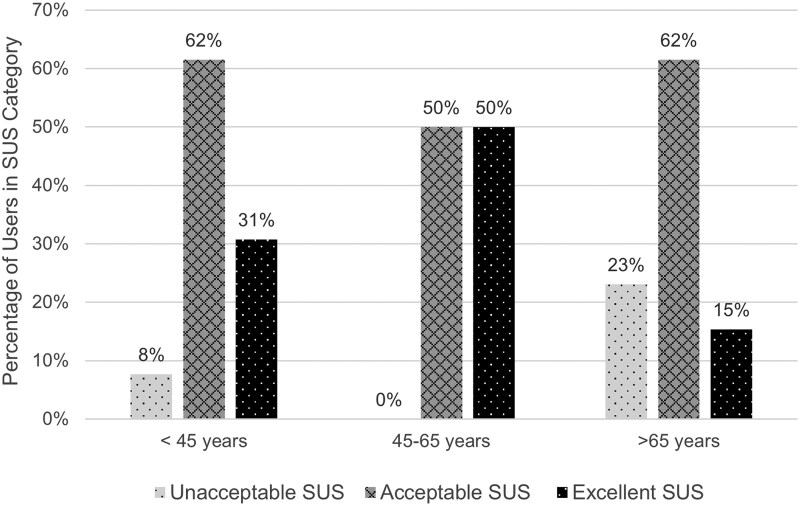
Age distribution by SUS score category (N = 32). Abbreviation: SUS, System Usability Scale.

**Figure 3.**
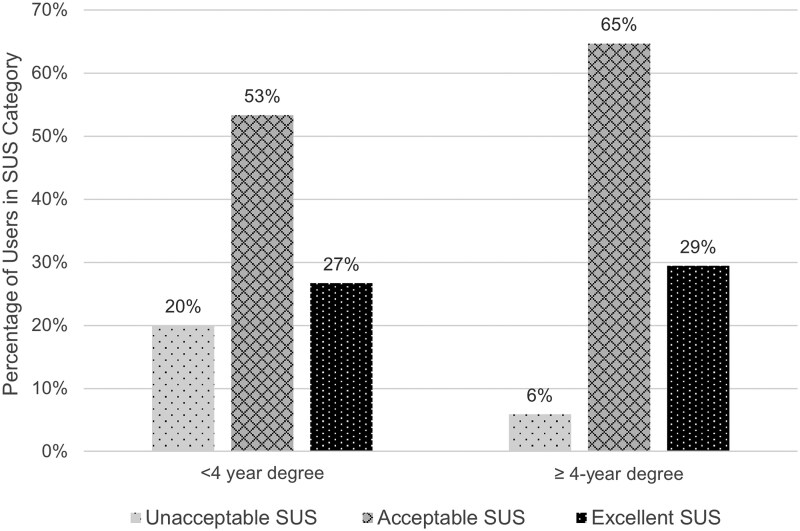
Education level by SUS score category (N = 32). Abbreviation: SUS, System Usability Scale.

Overall, the SUS scores were largely acceptable (20/43, 47%) or excellent (15/43, 35%) (Table 2). In Phase 1, a higher proportion of users had unacceptable SUS responses (4/11, 36%) compared to Phase 2 (4/32, 12.5%). Analysis of Phase 1 SUS responses by item showed the lowest mean score for question 5 (“I found the various functions in this system were well integrated.”) (Table 3). Among Phase 2 users, Fisher exact testing showed no statistically significant association (*P* = .51) between SUS score category (unacceptable, <65; acceptable, 65 ≤ score <85; or excellent ≥85) and age group (<45 years, 45-65 years, >65 years) (Fig. 2) or significant association (*P* = .53) between SUS score category and highest education level (< or ≥ 4-year degree) (Fig. 3). There was also no significant association (*P* = .06) between SUS-category and component selection (physical kit + paper guide, physical kit + website).

**Table 2.** User socio-demographics of interest (age, education level) by SUS score category (N = 43)

	Unacceptable SUS (% column)	Acceptable SUS (% column)	Excellent SUS (% column)
**Number of users**	8	20	15
**Age, years**			
Mean (std)	51.6 (25.9)	48.4 (21.4)	40.0 (19.5)
Median	52	57.5	32
<45 years	4 (50%)	9 (45%)	9 (60%)
45-65 years	1 (12%)	3 (15%)	3 (20%)
>65 years	3 (38%)	8 (40%)	3 (20%)
**Highest education level:**			
<4-year degree	3 (38%)	8 (40%)	4 (27%)
**≥**4-year degree	5 (62%)	12 (60%)	11 (73%)

Abbreviation: SUS, System Usability Scale.

**Table 3.** Phase 1 SUS survey results (N = 11)

	N	Excellent SUS (% row)	Acceptable SUS (% row)	Unacceptable SUS (% row)
Overall	11	6 (55%)	1 (9%)	4 (36%)

SUS question	User response category counts (% row)					Mean response*^b^*
	Strongly positive*^a^*	Positive	Neutral	Negative	Strongly negative	
**Q1:** I think that I would like to use this system frequently	3 (27%)	5 (45%)	1 (9%)	1 (9%)	1 (9%)	3.7
**Q2:** I found the system unnecessarily complex	5 (45%)	3 (27%)	2 (18%)	1 (9%)	0 (0%)	4.1
**Q3:** I thought the system was easy to use	4 (36%)	3 (27%)	4 (36%)	0 (0%)	0 (0%)	4.0
**Q4**: I think that I would need the support of a technical person to be able to use this system	9 (82%)	2 (18%)	0 (0%)	0 (0%)	0 (0%)	4.8
**Q5:** I found the various functions in this system were well integrated	2 (18%)	4 (36%)	3 (27%)	2 (18%)	0 (0%)	3.5
**Q6:** I thought there was too much inconsistency in this system	4 (36%)	5 (45%)	2 (18%)	0 (0%)	0 (0%)	4.2
**Q7:** I would imagine that most people would learn to use this system very quickly	4 (36%)	4 (36%)	1 (9%)	2 (18%)	0 (0%)	3.9
**Q8:** I found the system very cumbersome to use	7 (64%)	0 (0%)	2 (18%)	2 (18%)	0 (0%)	4.1
**Q9:** I felt very confident using the system	3 (27%)	6 (55%)	2 (18%)	0 (0%)	0 (0%)	4.1
**Q10:** I needed to learn a lot of things before I could get going with this system	8 (73%)	1 (9%)	1 (9%)	0 (0%)	1 (9%)	4.4

Abbreviation: SUS, System Usability Scale.

^a^A strongly positive response corresponds to a response of “strongly agree” for odd numbered questions (i.e Q1, Q3, …) and “strongly disagree” for even numbered questions (i.e Q2, Q4, …) on the SUS questionnaire.

^b^Mean was calculated by enumerating users' responses as follows: strongly negative response = 1, negative response = 2, neutral response = 3, positive response = 4, strongly positive response = 5.

### Skills Testing

Video analysis of Phase 2 users (N = 32) (Table 4) showed successful insulin injection in all users, regardless of SUS score category. Most (29/32, 91%) users successfully simulated use of the lancing device. Of the 3 users who were unable to complete this task, 1 (33%) had an acceptable SUS score and 2 (66%) had excellent scores. In addition, 28/32 (88%) users completed the test independently. Similarly, SUS scores remained acceptable (3/4, 75%) or excellent (1/4, 25%) for users who did not complete the skills testing independently. Finally, 23/32 (72%) users executed the steps needed to complete each skill in the recommended order. Of the 9 users who did not execute the steps of the skill in the recommended order, 2 (22%) had an unacceptable SUS score, 4 (44%) had an acceptable score and 3 (33%) had an excellent score.

**Table 4.** Phase 2 skills attainment results by SUS score category (N = 32)

	Overall (% column)	Unacceptable SUS (% row) (% column)	Acceptable SUS (% row) (% column)	Excellent SUS (% row) (% column)
**Number of users**	32	4 (13%) -	19 (59%) -	9 (28%) -
**Successful simulated insulin injection**				
Yes	32 (100%)	4 (13%) (100%)	19 (59%) (100%)	9 (28%) (100%)
No	0 (0%)	0 - (0%)	0 - (0%)	0 - (0%)
**Successful simulated lancing device use**				
Yes	29 (91%)	4 (14%) (100%)	18 (62%) (95%)	7 (24%) (78%)
No	3 (9%)	0 - (0%)	1 (33%) (6%)	2 (67%) (22%)
**Completed test independently**				
Yes	28 (87%)	4 (14%) (100%)	16 (57%) (84%)	8 (29%) (89%)
No	4 (12%)	0 (0%) (0%)	3 (75%) (16%)	1 (25%) (11%)
**Followed recommended educational pathway**				
Yes, followed	23 (72%)	2 (9%) (50%)	15 (65%) (79%)	6 (26%) (67%)
No, deviated	9 (28%)	2 (22%) (50%)	4 (44%) (21%)	3 (33%) (33%)

Abbreviation: SUS, System Usability Scale.

Skills testing outcomes among Phase 2 users were comparable between paper guide and website users. Among paper guide users, 16/17 (94%) successfully simulated lancing device use, 15/17 (88%) users completed skills testing independently, and 12/17 (71%) completed the skill in the recommended order compared to 13/15 (87%), 13/15 (87%), and 11/15 (73%) for each skill testing outcome respectively among website users. The steps at which user failures (ie, performed skill incorrectly, required facilitator assistance, or deviated from the educational pathway) occurred varied widely among users. However, among Phase 2 paper guide users with any failure, 3 of 5 (60%) were noted to have failures during steps related to loading the lancing device. Whereas, among Phase 2 website users with any failure, 1 of 4 (25%) had failures during lancing device loading.

### User Selection of Website or Paper Guide

More Phase 2 users primarily used the paper guide (17/32, 53%) with the physical toolkit than the website (15/32, 47%)(Table 1). The mean age of paper guide users (53.9 years) was older than website users (45.9 years). Many website users were <45 years old (7/15, 47%), whereas most paper guide users were >65 years old (9/17, 53%).

## Discussion

This study describes using a hybrid usability and skills testing protocol to evaluate a novel multi-modal, multi-component Diabetes Survival Skills Toolkit for DM self-care education. The high rate of successful attainment of skills in insulin injection (100%) and lancing device use (91%) among adult users of diverse ages and education levels reinforces the advantages of using a UCD approach to create and test DM self-care education interventions. Usability scores of the website/paper guide were overwhelmingly acceptable and excellent; however, they demonstrated greater variability than skills testing results. Usability scores were not associated with age-category or highest education level, nor were they correlated with skills attainment. Somewhat surprisingly, suboptimal skills attainment (eg, unsuccessful use of lancet, inability to navigate learning process independently) was not correlated with lower usability scores (unacceptable SUS).

### Toolkit Usability and Skills Attainment

After using the component(s) of the Kit, most users rated the usability as acceptable or excellent and successfully attained target DM survival skills. Ensuring DM education interventions impart survival skills needed to identify and prevent glycemic emergencies is particularly important given the persistently high rates of hypoglycemic and hyperglycemic emergencies among US adults [25], most frequently caused by insulin errors and omissions respectively [26, 27]. The stable rates of glycemic emergencies despite recent innovations in DM management also point to a translational challenge in changing behaviors and outcomes outside of controlled, hospital settings. While further testing is needed to investigate the effects of Kit delivery on clinical DM outcomes, this study's usability testing results show that users found the Kit highly engaging, which supports the Kit's promise to be adopted and promote change in real-world settings.

Additionally, the results of this study show that the Kit's usability scores did not differ significantly by age group or highest education level category. Age and educational level have both been previously associated with different educational preferences and barriers to DM education delivery, particularly in remote settings [7, 28 ]. Offering users the option to use the website or the paper guide to learn DM survival skills may influence the success of the Kit by better accommodating different user preferences. Phase 2 paper guide users had a higher mean age and proportion of users >65 years old compared to website users, yet SUS and skills testing outcomes were similar between paper guide and website users. These results, combined with growing evidence of the benefits of remote DM care delivery (ie, increased time and cost savings for patients and providers, reduced geographic and logistical barriers to care [4, 29, 30]), ultimately speak to the potential of UCD methods and this novel Kit to improve accessibility, equity, and efficiency of DM education delivery.

### Value of Both Usability and Survival Skills Testing

The study findings suggest that testing both usability *and* skill attainment together is advantageous compared to using either method alone. If only usability was tested, the failure of several Phase 2 users to successfully attain the lancet skill would not have been detected. It is possible these users' SUS scores may have been inflated by misconceptions that they performed the task correctly, given that facilitators did not provide feedback to users on if they performed a skill correctly during testing. Further, all of the Phase 2 users with unacceptable SUS scores still demonstrated successful attainment of the lancet and insulin injection skills as well as completed skills testing independently. Interpreting these results as purely design failures would be misleading. However, it highlights how successful skills completion, while important to clinicians and educators' perceptions of intervention usability, may be less important determinants of user's usability perception compared to other potential factors such as amount of time spent or cognitive effort required.

Alternatively, if the study were to have only conducted skills testing, important information about user assessment of the website/paper guide would be missed. The results of the study would have shown very high levels of skill attainment and might lead to premature closure/acceptance of a design, despite nearly 20% of users having unacceptable SUS scores.

The next step of the UCD process is to utilize the results of both usability and skills attainment testing to further refine the Kit. For example, since SUS item #5 (“I found the various functions in this system were well integrated”) had the lowest mean score among Phase 1 users, we refined and clarified how the components of the Kit are connected and used together before Phase 2 tests. As a result, item #5 was found to have the highest average response among Phase 2 SUS users who utilized all the Kit components together (Supplementary Table S1) [16]. In Phase 2, skills testing helped to identify at what step failures occurred. For example, a high number of failures were observed while loading the lancing device using the paper guide instructions. Skills testing can thus complement usability testing to better identify priorities for design improvement. This dual testing is highly recommended for the design and development of future digital and hybrid interventions.

### Study Strengths and Limitations

The strengths of this study are in its application of UCD and rigorous user testing methods to evaluate a novel Diabetes Survival Skills Toolkit. The use of SUS scores in combination with skill achievement results provides unique depth in understanding the usability of the toolkit.

There are, however, limitations to the study. Nonrandom sampling of our local networks was used. We acknowledge this could produce some meaningful differences between the study population and the target users of Kit (patients with DM newly prescribed insulin). However, the study's exclusion criteria and purposive sampling resulted in a pragmatic sample of users who were unfamiliar with DM self-care and who varied in demographic factors known to influence DM education delivery. Thus, user testing results from this sample still hold significant interest in informing the design, usability, and efficacy of the Kit for the purposes of this study. Expanding testing to create a sample more representative of DM patients with respect to race and ethnicity could improve generalizability [31, 32].

## Conclusions

The Diabetes Survival Skills Toolkit demonstrated high DM survival skills attainment when tested in a sample of layperson users, diverse in age and education levels. In contrast, perceived usability of the Kit varied and was not correlated with skill attainment. These findings underscore that perceived usability and skills attainment are different concepts and should both be tested. Conducting both usability and skill testing can optimize the design of DM self-care education interventions. This study also provides evidence that remote testing of both usability and skills attainment is a highly feasible and reliable testing modality.

## Data Availability

Restrictions apply to the availability of all data generated or analyzed during this study to preserve participant confidentiality. The corresponding author will on request detail the restrictions and any conditions under which access to some data may be provided.

## Abbreviations

DM: diabetes mellitus
Kit: Diabetes Survival Skills Toolkit
SUS: System Usability Scale
UCD: user-centered design
